# Characterisation of transcription factor profiles in polycystic kidney disease (PKD): identification and validation of STAT3 and RUNX1 in the injury/repair response and PKD progression

**DOI:** 10.1007/s00109-019-01852-3

**Published:** 2019-11-26

**Authors:** Chiara Formica, Tareq Malas, Judit Balog, Lotte Verburg, Peter A. C. ‘t Hoen, Dorien J. M. Peters

**Affiliations:** 1 Department of Human Genetics, Leiden University Medical Center, Einthovenweg 20, 2333 ZC Leiden, The Netherlands; 2 Department of Pathology, Leiden University Medical Center, Albinusdreef 2, 2333 ZA Leiden, The Netherlands; 3 Centre for Molecular and Biomolecular Informatics, Radboud Institute for Molecular Life Sciences, Radboud University Medical Center Nijmegen, Geert Grooteplein Zuid 26/28, 6525 GA Nijmegen, The Netherlands

**Keywords:** Autosomal dominant polycystic kidney disease, kidney injury, Gene expression, Transcription factors, Chromatin immunoprecipitation

## Abstract

**Abstract:**

Autosomal dominant polycystic kidney disease (ADPKD) is the most common genetic renal disease, caused in the majority of the cases by a mutation in either the *PKD1* or the *PKD2* gene. ADPKD is characterised by a progressive increase in the number and size of cysts, together with fibrosis and distortion of the renal architecture, over the years. This is accompanied by alterations in a complex network of signalling pathways. However, the underlying molecular mechanisms are not well characterised. Previously, we defined the PKD Signature, a set of genes typically dysregulated in PKD across different disease models from a meta-analysis of expression profiles. Given the importance of transcription factors (TFs) in modulating disease, we focused in this paper on characterising TFs from the PKD Signature. Our results revealed that out of the 1515 genes in the PKD Signature, 92 were TFs with altered expression in PKD, and 32 of those were also implicated in tissue injury/repair mechanisms. Validating the dysregulation of these TFs by qPCR in independent PKD and injury models largely confirmed these findings. STAT3 and RUNX1 displayed the strongest activation in cystic kidneys, as demonstrated by chromatin immunoprecipitation (ChIP) followed by qPCR. Using immunohistochemistry, we showed a dramatic increase of expression after renal injury in mice and cystic renal tissue of mice and humans. Our results suggest a role for STAT3 and RUNX1 and their downstream targets in the aetiology of ADPKD and indicate that the meta-analysis approach is a viable strategy for new target discovery in PKD.

**Key messages:**

- We identified a list of transcription factors (TFs) commonly dysregulated in ADPKD.
- Out of the 92 TFs identified in the PKD Signature, 35% are also involved in injury/repair processes.
- STAT3 and RUNX1 are the most significantly dysregulated TFs after injury and during PKD progression.
- STAT3 and RUNX1 activity is increased in cystic compared to non-cystic mouse kidneys.
- Increased expression of STAT3 and RUNX1 is observed in the nuclei of renal epithelial cells, also in human ADPKD samples.

**Electronic supplementary material:**

The online version of this article (10.1007/s00109-019-01852-3) contains supplementary material, which is available to authorized users.

## Introduction

Autosomal dominant polycystic kidney disease (ADPKD) is a genetic disease characterised by the formation of fluid-filled renal cysts. Cyst formation and cyst growth are accompanied by inflammation and fibrosis, leading to kidney failure. In the majority of cases, ADPKD is caused by a mutation in the *PKD1* gene or, less frequently, in the *PKD2* gene. Nevertheless, ADPKD is a complex disease which involves the dysregulation of many different signalling pathways [1], and the molecular mechanisms involved in disease progression are not entirely understood. Currently, the vasopressin V2-receptor antagonist, tolvaptan, is the only approved treatment in Europe but only for selected patients. More generic and definitive treatment is still missing.

Both environmental and genetic factors can be considered disease modifiers in ADPKD [1, 2]. An important one is renal injury, shown to accelerate cyst formation and expansion in different mouse models [3, 4]. Recently, we showed that renal injury shares molecular processes with ADPKD progression. Using a meta-analysis approach, we identified a set of genes dysregulated in a variety of PKD models during disease progression, which we called the “PKD Signature”. About 35% of these genes were found to be also implicated in injury/repair mechanisms, confirming the strong relation between ADPKD and injury [5].

Transcription factor (TF) proteins are master regulators of transcription, which control the expression of genes involved in the establishment and maintenance of cell states, in physiological and pathological situations. Dysregulation of TFs levels and/or activity can lead to the development of a broad range of diseases. Thus, identification of a TFs profile in ADPKD could help to better understand the molecular mechanisms contributing to cyst formation. For this reason, in this study, we focus on the signature of TFs. We identified new PKD-related TFs, and we validated altered expression during ADPKD progression and injury/repair in different mouse models. For two of the identified TFs, STAT3 and RUNX1, we also showed increased activity in mouse cystic kidneys, as well as altered expression in human ADPKD kidneys.

## Materials and methods

### Identification of transcription factors in PKD

Identification of the PKD Signature was described previously [5]. Briefly, in the previous work, we performed a meta-analysis of PKD expression profiles across different disease models and identified 1515 genes that showed consistent dysregulation across the different PKD studies. We further identified genes involved in injury/repair processes from the PKD Signature by firstly producing injury repair gene profile based on several injury-induced animal models and secondly intersecting the identified PKD Signature and injury repair profiles for the identification of overlapping genes.

In this publication, we used MSigDB’s collection of TFs based on Messina et al*.* [6] and Moreland et al. [7] for the identification of TFs involved in PKD. Furthermore, we identified the transcription factors that are involved in the injury/repair processes of PKD based on the previously identified injury repair profile [5].

The enrichment of TF targets in the PKD Signature was based on the target collections in the ChEA 2016 database [8] that includes TF targets based on experimental evidence. We calculated the enrichment using the representation factor method described below. TFs are considered enriched if they had a representation factor above 1. The representation factor is the number of overlapping genes divided by the expected number of overlapping genes drawn from two independent groups. A representation factor > 1 indicates more overlap than expected of two independent groups, and a representation factor < 1 indicates less overlap than expected. The formula used to calculate the representation factor is x/(n × D)/N, where x = # of genes in common between two groups; *n* = # of genes in group 1 (the total number of targets calculated per transcription factor based on ChEA 2016 database); D = # of genes in group 2 (the total number of genes in the PKD Signature up (775) or down (740) regulated lists independently); *N* = total genes, in this case, the 10,271 genes with Entrez IDs.

### In silico functional annotation of gene lists

GeneTrail2 v1.6 [9] was used to identify the enriched/significant pathways/functions of the identified gene lists. For all analyses, we used Wikipathways as the primary source of annotation. GeneTrail2 v1.6 was run with the following parameters: overrepresentation analysis (enrichment algorithm); FDR adjustment (adjustment method); significance level at 0.05; and minimum and maximum size of the category equal to 2 and 700, respectively.

### Gene expression and statistical analysis of the significance of results

Snap-frozen mouse kidneys were homogenised using MagNa Lyser technology (Roche). Total RNA was isolated using TRI Reagent (Sigma-Aldrich). cDNA synthesis was performed using Transcriptor First Strand cDNA Synthesis Kit (Roche), and qPCR was done using 2× FastStart SYBR Green Master (Roche) according to the manufacturer’s protocol. Alternatively, it was performed at GenomeScan (GenomeScan B.V.) using the 96.96 BioMark™ Dynamic Array for real-time PCR (Fluidigm Corporation), as previously described [5]. Gene expression was normalised to the geometric mean of three housekeeping genes (*Rplp0, Hnrnpa2b1, Ywhaz*) for Fluidigm data and *Hprt* for SYBR-Green data. The output of the Fluidigm assay was normalised and converted into Ct values (cycle threshold). For each transcription factor, a two-way ANOVA was conducted to compare the genotype (PKD vs WT) and the treatment (PBS vs DCVC) effects for each age-matched time points. The computation was made using the *Limma package* [10] in R. A list of primer sequences and TaqMan assays can be found in Supplementary Table 3.

### Identification of transcription factors binding sites and primer design

For the TFs that were selected for our ChIP analysis, we identified the binding sites of each TF and its targets by screening the Cistrome database [11] and accessing all studies that performed ChIP-Seq experiments on our selected TFs. We looked for peaks that appeared with an intensity of 10 or higher in more than one ChIP-Seq study. We mapped the *Mus musculus* mm10 genome to the peaks identified using *Peak2Gene* tool that is part of the Cistrome Galaxy tools to identify genes that are within 10,000 base pairs of both ends of the peak. The peaks that did not map to a gene target that is part of the PKD Signature were eliminated. Finally, sorting on the intensity level of the peak, we visualised the top peaks on the UCSC Genome Browser [12] and selected the peaks that had sufficient height over noise levels for qPCR enrichment. We designed primers spanning the TFs binding sites on their putative target genes. The binding sites were generally overlapping with the promoter region of the target genes. As a negative control, we designed primers binding at about 5 kb from the promoter regions where we did not expect to find any TF-binding activity. A list of primers can be found in Supplementary Table 3. Two-way ANOVA with Tukey’s multiple comparisons test was performed comparing the input-normalised binding-enrichment of the TFs or the control IgG at the binding site and at the nonbinding sites.

### Animal model

All the animal experiments were evaluated and approved by the local animal experimental committee of the Leiden University Medical Center (LUMC) and the Commission Biotechnology in Animals of the Dutch Ministry of Agriculture. Kidney-specific tamoxifen-inducible *Pkd1*-deletion mouse model (iKsp*Pkd1*^del^) have been described previously [13]. We only used male mice, to reduce variability in disease progression as female mice tend to have a slower and milder progression of the disease compared to male mice [14]. Wt mice have only the LoxP sites around exons 2–11 of the *Pkd1* gene but not the Cre recombinase (*Pkd1*^loxlox^). For three consecutive days, 5 mg/kg of tamoxifen was administered via oral gavage when mice were 13–14 weeks old. Inactivation of the *Pkd1* gene at this age leads to cyst formation in all the renal tubule segments. A week later, mice were injected intraperitoneally with 15 mg/kg of the nephrotoxic compound S-(1,2-dichlorovinyl)-L-cysteine (DCVC) or vehicle (PBS) as a control. Kidney function was evaluated using blood urea nitrogen (BUN) level as previously described [4]. Renal failure is defined by BUN equal or higher than 25 mmol/l. Mice were sacrificed at 1, 2, 5 and 10 weeks after DCVC and kidney failure. The experimental pipeline has been presented in Formica et al. [15]. The Wt + PBS, Wt + DCVC and *Pkd1* KO + PBS groups have also been used in Malas et al. [5]. At the sacrifice, kidneys were collected and weighed. For RNA and chromatin extraction, kidneys were snap frozen in liquid nitrogen. For immunohistochemistry (IHC) staining, kidneys were preserved in phosphate-buffered 4% formaldehyde solution. A t-test was conducted to compare median survival in PBS-treated versus DCVC-treated mice and BUN in Wt versus iKsp*Pkd1*^del^ mice.

### ChIP

Chromatin was isolated from mouse inner-medullary collecting duct (mIMCD3; ATCC, Rockville, USA) cells (about 5 × 10^6^/ml). Briefly, cells were crosslinked with 1% formaldehyde for 10 min at RT then lysed with buffer with protease and phosphatase inhibitors (Roche) as described on Nature protocols (ChIP buffer) [16].

For kidneys’ chromatin extraction, snap-frozen kidneys, harvested at end-stage renal disease (ESRD) from Wt mice and iKsp*Pkd1*^del^ mice treated with DCVC or PBS, were cut with a blade in a petri dish then fixed with 1% formalin (50 mg/ml) rocking for 12 min at RT. Glycine (0.125 M) was added to stop the reaction, and the tissue was washed with PBS with serine protease inhibitor phenylmethylsulfonyl fluoride (PMSF). The tissue was resuspended in cytoplasmic lysis buffer and moved in a glass tissue grinder (Kimble Chase) for homogenisation and then filtered using a 50 μm filter (CellTrics® Sysmex). The homogenate was washed and then lysed with ChIP buffer with protease and phosphatase inhibitors. Chromatin was sonicated in ChIP buffer using a Diagenode Bioruptor® Pico (Diagenode) 30 s on/30 s off for 15 cycles. Fragment size was checked by gel electrophoresis.

For immunoprecipitation, 60 μg of chromatin were used per reaction. Sepharose protein A alone or mixed 4:1 with protein G (GE Healthcare) were used to preclear the chromatin before incubation with primary antibodies for 4 h at 4 °C. Primary antibodies used 5 μg rabbit anti-pSTAT3 (Cell Signalling #9145); 8 μg mouse anti-RUNX1 (Santa Cruz Biotechnology, Inc. #sc-365644); rabbit anti-IgG (Abcam #ab37415) and mouse anti-IgG (Cell Signalling #5415S). 20 μl of Sepharose protein A (for pSTAT3) or A/G 4:1 (for RUNX1) were added to each sample and incubated overnight at 4 °C. Samples were collected by centrifugation and washed with low-salt wash buffer (150 mM NaCl, 20 mM Tris-HCl pH 8.1, 2 mM EDTA, 0.1% SDS, 1% Triton X-100), high-salt wash buffer (500 mM NaCl, 20 mM Tris-HCl pH 8.1, 2 mM EDTA, 0.1% SDS, 1% Triton X-100), LiCl wash buffer (10 mM Tris-HCl pH 8.1, 1 mM EDTA, 0.25 M LiCl, 1% NP-40, 1% sodium deoxycholate) and twice with TE wash buffer (10 mM Tris-HCl pH 8.1, 1 mM EDTA). Cross-links were reversed incubating with Chelex®100 resin beads (Bio-Rad #142-1253) at 99 °C for 15 min on a shaking block, and then the samples were diluted 1:1 with MQ water.

### IHC

Kidneys fixed in formalin and embedded in paraffin were cut at 4 μm thickness. Sections were stained with the primary antibodies used for ChIP: rabbit anti-pSTAT3 (1:75; Cell Signalling #9145) and mouse anti-RUNX1 (1:250; Santa Cruz Biotechnology, Inc. #sc-365644). Anti-rabbit or anti-mouse Envision HRP (Dako) was used as the secondary antibody.

Renal tissue from ADPKD patients at end-stage renal failure was fixed in formalin as previously described [15]. Control tissues were obtained from donor kidneys non-suitable for transplant. All human tissue samples were collected following procedures approved by the LUMC medical ethical committee (institutional review board).

## Results

### Transcription factors in the PKD signature

Using a meta-analysis approach of published PKD expression profiles and in-house generated RNA-sequencing data on our *Pkd1* mutant mouse model (iKsp*Pkd1*^del^), we recently identified 1515 genes that are commonly dysregulated across several PKD disease models, hereafter referred to as the PKD Signature [5].

**Fig. 1:**
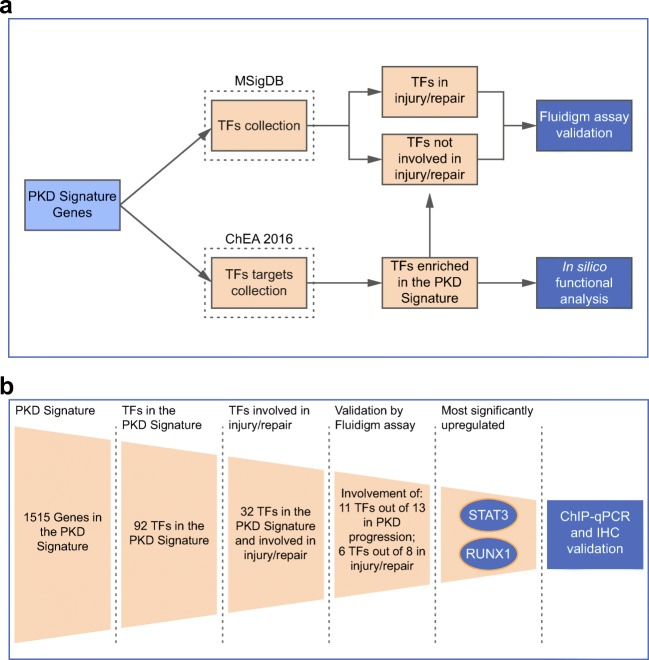
Schematic representation of the workflow used for the identification and validation of TFs involved in PKD and injury/repair. **a** MSigDB was used to select the TFs in the PKD Signature. ChEA 2016 was used to select the TFs with most deregulated, experimentally verified targets in the PKD Signature (note: the ChIP-chip and ChIP-Seq experiments in ChEA 2016 were typically from cell lines not necessarily related to the kidney). The TFs identified with MSigDB in the PKD Signature were intersected with the injury signature generated in our previous work [5] to obtain TFs involved in injury/repair mechanism, and TFs involved only in PKD progression. Fluidigm assay was used to validate the expression of selected TFs identified by this analysis. The TFs identified based on their target genes using the ChEA 2016 database were intersected with the TFs identified in the PKD signature to identify the overlapping TFs. In silico pathway analysis was performed on the overlapping TFs and their target genes to identify significant pathways modulated by the TFs. **b** Schematic representation of the workflow used to identify and validate selected TFs. The two most significant TFs identified were STAT3 and RUNX1 which were further investigated in cystic kidneys using chromatin immunoprecipitation-qPCR (ChIP-qPCR) and immunohistochemistry (IHC)

We used MSigDB to identify the TFs that are part of the PKD Signature (Fig 1a). Out of the 1515 genes of the PKD Signature, we identified 92 TFs that were differentially expressed and could be involved in cyst formation and PKD development. Among the 92 TFs identified, 32 were also implicated in tissue injury/repair mechanisms based on our previously defined injury repair profile (Supplementary Table 1) [5]. Several of the herein identified TFs, such as STAT3 and MYC, are known players in ADPKD progression [17, 18]. Nevertheless, many others have never been described in ADPKD before.

Furthermore, we predicted TFs that are relevant to PKD based on the enrichment of their targets in the PKD Signature. Using the ChEA 2016 database of TF targets, we identified TFs with more experimentally verified targets (ChIP-chip or ChIP-Seq) overlapping with the PKD Signature than would be expected by chance (Fig. 1a). The TFs E2F7, TRIM28, TP63 (two different experiments in different cell lines), EGR1 and STAT3 were most significant in this analysis (Supplementary Table 2a) since targets of these TFs were mostly upregulated in PKD. Five TFs were both in the list of TFs identified based on their targets and among the 92 TFs present in the PKD Signature: EGR1, ESR1, STAT3, FOXM1 and KLF5. Thus, these TFs, as well as their identified direct targets, were dysregulated in PKD (Supplementary Table 2b). Further pathway analysis of these five TFs targets uncovered involvement in the modulation of TGF-β signalling, estrogen signalling, apoptosis, oxidative stress, interleukins signalling, adipogenesis and cellular metabolism (Supplementary Table 2c).

### Validation of meta-analysis in independent samples

**Fig. 2:**
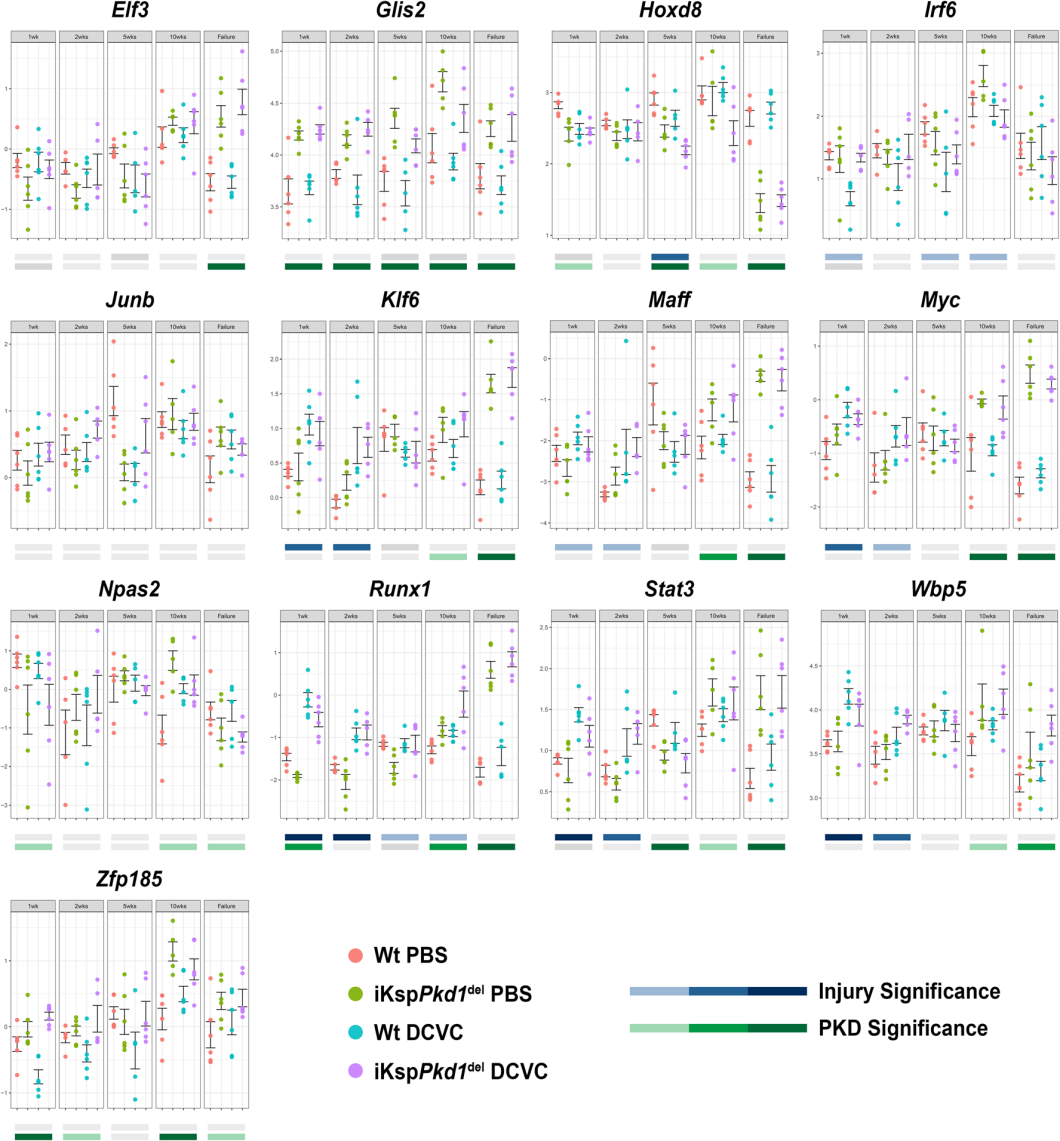
Expression of selected TFs using Fluidigm assay. TFs selected from the PKD Signature for experimental validation were subjected to qRT-PCR on RNA isolated from the kidneys of iKsp*Pkd1*^del^ mice and age-matched Wt mice at 1, 2, 5 and 10 weeks after DCVC and at kidney failure. On the Y-axis, normalized Ct values (cycle threshold values) are plotted for each gene separately across the five measurement time points for four types of samples: Wt mice treated with saline (Wt PBS, salmon), iKsp*Pkd1*^del^ mice treated with saline (iKsp*Pkd1*^del^ PBS, light green), Wt mice treated with DCVC (Wt DCVC, light blue) and iKsp*Pkd1*^del^ mice treated with DCVC (iKsp*Pkd1*^del^ DCVC, light purple). The analysis was based on comparing treatment (DCVC vs PBS) and genotype (iKsp*Pkd1*^del^ vs Wt) using a two-way ANOVA test. The resulting *P* values are shown with colour codes: darkest colour shade, *P* value < 0.0005; medium colour shade, *P* value < 0.005 and low colour shade at *P* value < 0.05. *P* value ≥ 0.05 were not considered significant (grey bars). Each dot is a mouse and whiskers reflect the mean ± SD. Expression of *Glis2* and *Stat3* in Wt PBS, iKsp*Pkd1*^del^ PBS and Wt DCVC have been published in Malas et al.(2017) [5].

Our next step was to validate TFs identified in the meta-analysis in independent experimental groups of mice during PKD progression and/or the nephrotoxic injury/repair response [15]. Briefly, we induced *Pkd1* deletion in adult mice via tamoxifen administration, which leads to a slow progression of the disease. Wild-type (Wt) mice received tamoxifen as well. A week after tamoxifen administration, we injected both genotypes with 15 mg/kg of DCVC, a nephrotoxic compound or PBS as a control. At this dosage, DCVC causes a repairable renal injury that is mostly recovered 1 to 2 weeks after injection but accelerates cyst formation resulting in tubular dilations at 10 weeks and renal failure around 14 weeks of age (Supplementary Fig. 1). Mice were sacrificed at 1, 2, 5 and 10 weeks after DCVC and at kidney failure. Kidneys harvested at these time points were used to evaluate gene expression of selected TF using the Fluidigm qPCR chip (Fig. 1b). Out of the 92 TFs, 13 were selected for further analysis, based on transcript levels, altered expression in the injury/repair response and involvement in multiple molecular pathways (Supplementary Table 1). In our Fluidigm setup, we had four groups: PBS-treated Wt, DCVC-treated Wt, PBS-treated iKsp*Pkd1*^del^ and DCVC-treated iKsp*Pkd1*^del^ at five time points (1week, 2weeks, 5weeks and 10weeks after DCVC treatment and at kidney failure). Out of the 13 tested TFs, 11 were significantly different (*P* < 0.05) in PKD samples compared to Wt, while the involvement of *Irf6* and *JunB* could not be confirmed (Supplementary Table 1, Fig. 2). We also evaluated whether expression of the 13 TFs was affected by injury, by comparing DCVC versus PBS-treated animals at injury-related time points (1week, 2weeks and 5weeks after DCVC treatment). Of the 13 selected TFs, 8 were part of the previously reported injury repair profile, while 5 were not [5]. We confirmed significant injury-induced dysregulation (*P* < 0.05) of 6 out of 8 TFs predicted to be involved in the injury/repair mechanism by the meta-analysis, while we did not see any significant dysregulation of the expression of 3 out of 5 TFs that were not found in the meta-analysis (Supplementary Table 1, Fig. 2) [5]. Notably, the expression of *Runx1* and *Stat3* was most significantly affected by DCVC-induced injury and PKD progression.

### Expression of two selected TFs in mouse kidneys during ADPKD progression and after injury

To further support the utility of meta-analysis approaches to new target discovery in ADPKD, we chose STAT3 and RUNX1 for additional experimental validation.

**Fig. 3:**
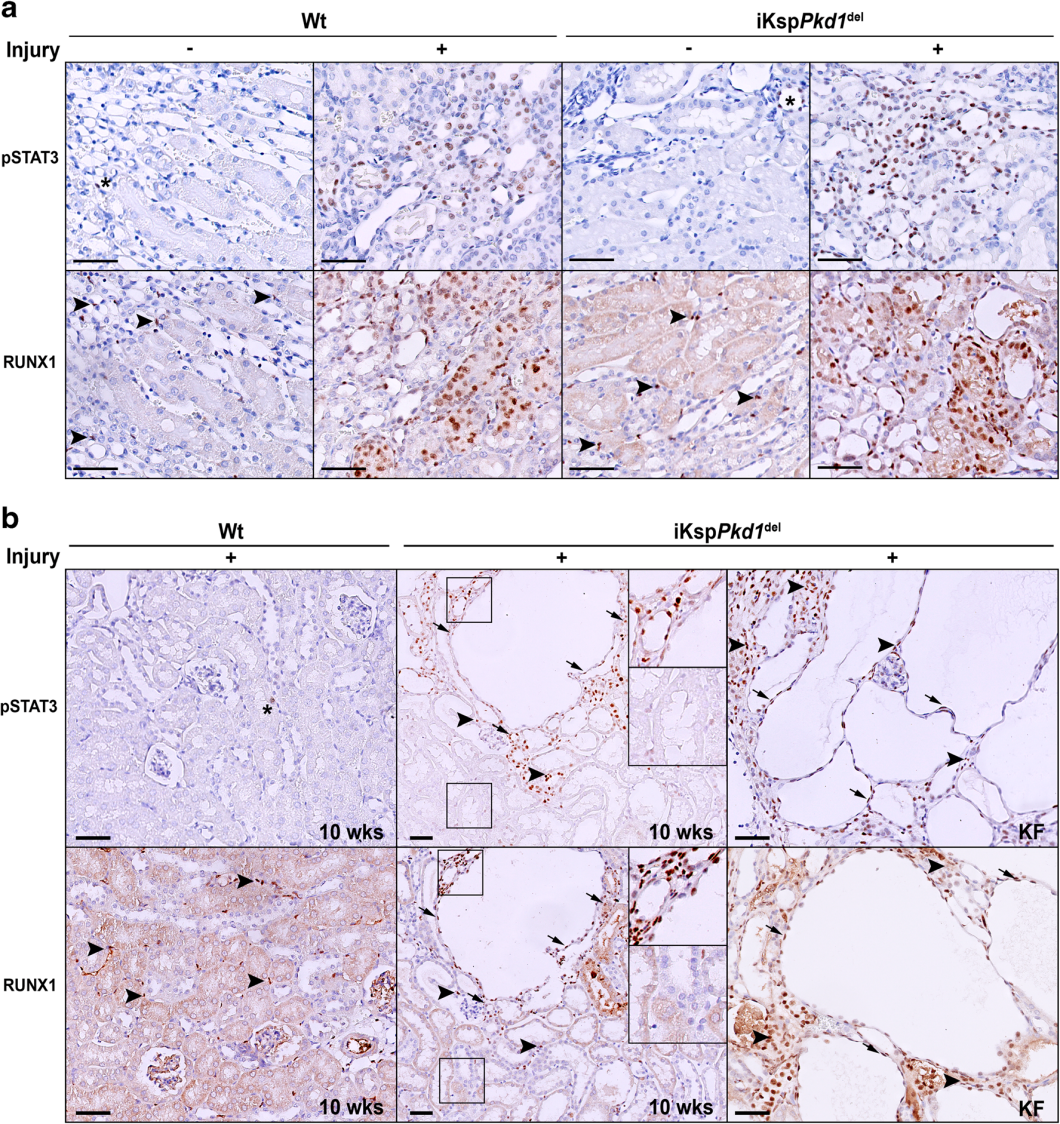
Expression of pSTAT3 and RUNX1 in Wt and iKsp*Pkd1*^del^ mice after injury and during cyst progression. **a** Representative immunohistochemistry of Wt and iKsp*Pkd1*^del^ kidneys at 1 week after DCVC (+ injury) or PBS (– injury). Mice without injury showed only sporadic expression of pSTAT3 in the nuclei of tubular epithelial cells (asterisks); after injury, the expression was markedly increased both in Wt mice and in iKsp*Pkd1*^del^ mice. RUNX1 expression in non-injured kidney was present only in some interstitial cells (arrowheads); after injury, RUNX1 was visible in the nuclei of the epithelial cells. **b** Representative immunohistochemistry of Wt and iKsp*Pkd1*^del^ kidneys at 10 weeks after DCVC (“10weeks”; left and middle panel) showed expression of pSTAT3 and RUNX1 in nuclei in cyst-lining epithelia, in the epithelial cells of surrounding dilated tubules (arrows) and in infiltrating cells (arrowheads) only in cystic tissue. Expression of pSTAT3 and RUNX1 was even more increased at kidney failure (“KF”; right panel) when the kidneys are severely cystic. Scale bars 50 μm

We performed immunohistochemical analysis for the active form of STAT3 (pSTAT3) and RUNX1 and studied activation and subcellular localisation. In non-injured Wt and iKsp*Pkd1*^del^ mice, pSTAT3 and RUNX1 are not detectable, except for some interstitial cells that show nuclear staining. Interestingly, after injury (at 1wk after DCVC), there was an intense nuclear expression of pSTAT3 and RUNX1 in both Wt and iKsp*Pkd1*^del^ mice (Fig. 3a and Supplementary Fig. 2a).

At 10 weeks post-DCVC, Wt mice have fully healed the renal damage and have largely pSTAT3 and RUNX1 negative kidneys, comparable to the Wt treated with PBS. Conversely, iKsp*Pkd1*^del^ mice, which already developed some mild cysts at this time point, showed expression of pSTAT3 and RUNX1 in the cyst-lining epithelial cells and some of the surrounding dilated tubules (Fig. 3b, middle panel and Supplementary Fig. 2b, middle panel). iKsp*Pkd1*^del^ mice treated with PBS, instead, have not undergone injury/repair phase nor displayed overt cyst formation at this time point and showed almost no expression of pSTAT3 and RUNX1, as expected.

At kidney failure, iKsp*Pkd1*^del^ mice present severe renal degeneration and cyst formation. At this time point, the expression of pSTAT3 and RUNX1 is markedly increased (Fig. 3b, right panel and Supplementary Fig. 2b, right panel). Interestingly, not only epithelial cells but also infiltrating cells stained positive for these TFs, suggesting that pSTAT3 and RUNX1 might be important in the regulation of signalling pathways in other cell types in addition to tubular epithelial cells (Fig. 3b, arrowheads).

In summary, we confirmed that pSTAT3 and RUNX1 protein expression were increased in the nuclei of tubular epithelial cells after injury and during PKD progression.

### Stat3 and Runx1 target genes were dysregulated during ADPKD progression and after injury

**Fig. 4:**
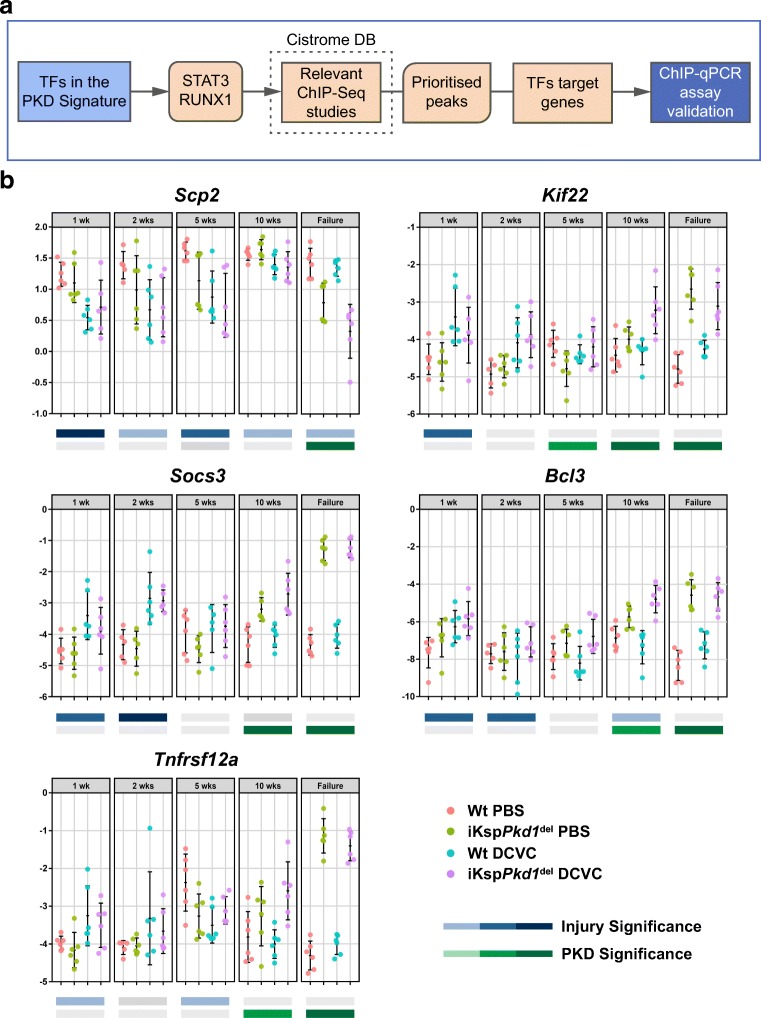
Identification of STAT3 and RUNX1 target genes. **a** STAT3 and RUNX1 emerged as two leading candidates for wet-lab validation. Using Cistrome database, we identified ChIP-peaks that were used in the wet-lab validation process and led to the identification of confirmed STAT3 and RUNX1 targets. **b** Expression of STAT3 and RUNX1 targets during PKD progression. Total RNA was isolated from kidneys of Wt and iKsp*Pkd1*^del^ mice treated with PBS or DCVC at 1, 2, 5 and 10 weeks and at kidney failure. Expression of selected STAT3 (*Scp2*, *Kif22* and *Socs3*) and RUNX1 (*Bcl3*, *Tnfrsf12a*) targets was evaluated using a SYBR Green-based qPCR. On the Y-axis, normalised Ct values (cycle threshold values) are plotted. Data were analysed using a two-way ANOVA test based on comparing treatment (DCVC vs PBS) and genotype (iKsp*Pkd1*^del^ vs Wt). *P* values are reported and classified into high significance (darkest colour shade) at *P* value < 0.0005, moderate significance (medium colour shade) at *P* value < 0.005 and acceptable significance at (low colour shade) at *P* value < 0.05. *P* value ≥ 0.05 was not considered significant (grey bars). Each dot is a mouse and whiskers represent mean ± SD

Although we demonstrated that pSTAT3 and RUNX1 expression were increased during ADPKD progression and after injury, both at gene and protein level, we do not know if this would translate into differences in their activity as transcriptional regulators. Thus, we quantified the expression of their target genes during PKD progression and injury/repair. To find TFs’ target genes, we used the publicly available Cistrome database. For both TFs, we identified ChIP-Seq experiments and searched for peaks (targets) identified in at least two ChIP-Seq experiments. Peaks were prioritised based on (1) the number of studies they were found in, (2) their intensity levels (> 10) and (3) whether they mapped to target genes within 10 kb distance. For both TFs, the top putative target genes were crossed with the PKD Signature genes to identify targets that show differential expression in PKD. Only target genes that were also present in the PKD Signature were selected for further analysis (Fig. 4a).

The final targets we selected are *Scp2*, *Kif22*, *Stat3* (autoregulation) and *Socs3* for STAT3 and *Runx1* (autoregulation), *Tnfrsf12a* and *Bcl3* as targets for RUNX1. We checked the expression of these targets after injury and during PKD progression in iKsp*Pkd1*^del^ and Wt mice. We found that, in iKsp*Pkd1*^del^ mice, all targets were significantly upregulated except for *Scp2*, which was downregulated, suggesting an inhibitory effect of STAT3 on *Scp2* transcription (Fig 2b, *Stat3* and *Runx1;* Fig. 4b, *Scp2*, *Kif22*, *Socs3, Tnfrsf12a* and *Bcl3*).

These data indicate that not only the level of expression of the selected TFs is dysregulated during injury/repair and PKD progression but likely also their activity, as denoted by the dysregulated expression of their target genes.

### Stat3 and Runx1 ChIP-qPCR in murine renal epithelial cells

**Fig. 5:**
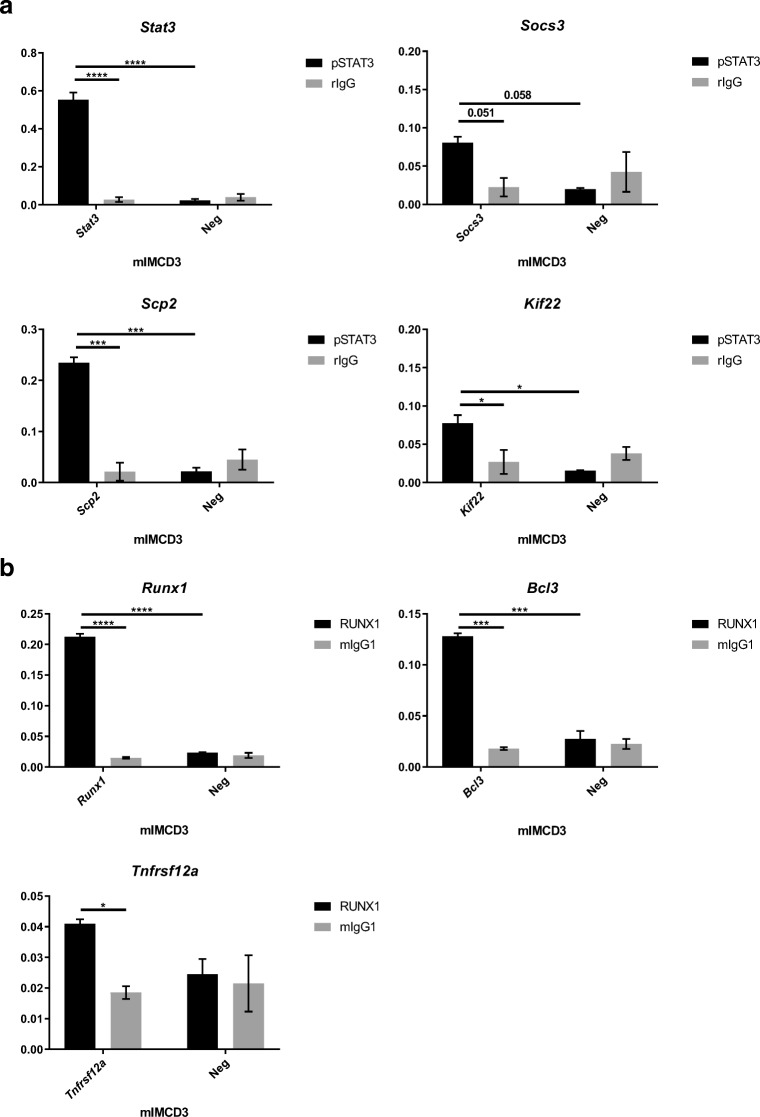
ChIP validation of pSTAT3 and RUNX1 targets in mIMCD3 cells. **a** ChIP with anti-pSTAT3 antibody showed significant enrichment at the promoter region of *Scp2*, *Kif22*, *Stat3* and *Socs3* compared to a negative control antibody (rIgG) and a nonbinding region (Neg). **b** ChIP with anti-RUNX1 antibody showed a significant enrichment at the promoter region of *Runx1*, *Tnfrsf12a* and *Bcl3* compared to a negative control antibody (mIgG) and a nonbinding region (Neg). The Y-axis shows the input-normalised binding-enrichment of the TFs to the indicated genomic region. Data represent the mean of two independent ChIPs ± SD; Two-way ANOVA with Tukey’s multiple comparisons test. *P value < 0.05; **P value < 0.01; ***P value < 0.001

To confirm that STAT3 and RUNX1 are directly regulating the expression of the indicated target genes in the renal epithelium, we performed chromatin immunoprecipitation (ChIP) analysis followed by quantitative PCR (ChIP-qPCR). We first confirmed that STAT3 and RUNX1 were expressed in mIMCD3 cells (Supplementary Fig 3). We then isolated chromatin and performed ChIP-qPCR. STAT3 enrichment at the promoter region of the *Scp2*, *Kif22*, *Stat3* and *Socs3* genes was significantly higher than at nonbinding regions (Fig. 5a). Also, RUNX1 showed significant enrichment at the promoter regions of its targets *Runx1*, *Tnfrsf12a* and *Bcl3* (Fig. 5b) compared to nonbinding regions.

Thus, we can conclude that STAT3 and RUNX1 are actively binding the selected target genes in renal epithelial cells.

### Stat3 and Runx1 ChIP-qPCR in murine kidney tissue

We then investigated whether binding of STAT3 and RUNX1 at the promoter region of their target genes is increased in cystic kidneys compared to non-cystic kidneys. To do so, we performed ChIP-qPCR using kidneys from iKsp*Pkd1*^del^ mice, harvested at kidney failure, as well as age- and treatment-matched Wt kidneys.

**Fig. 6:**
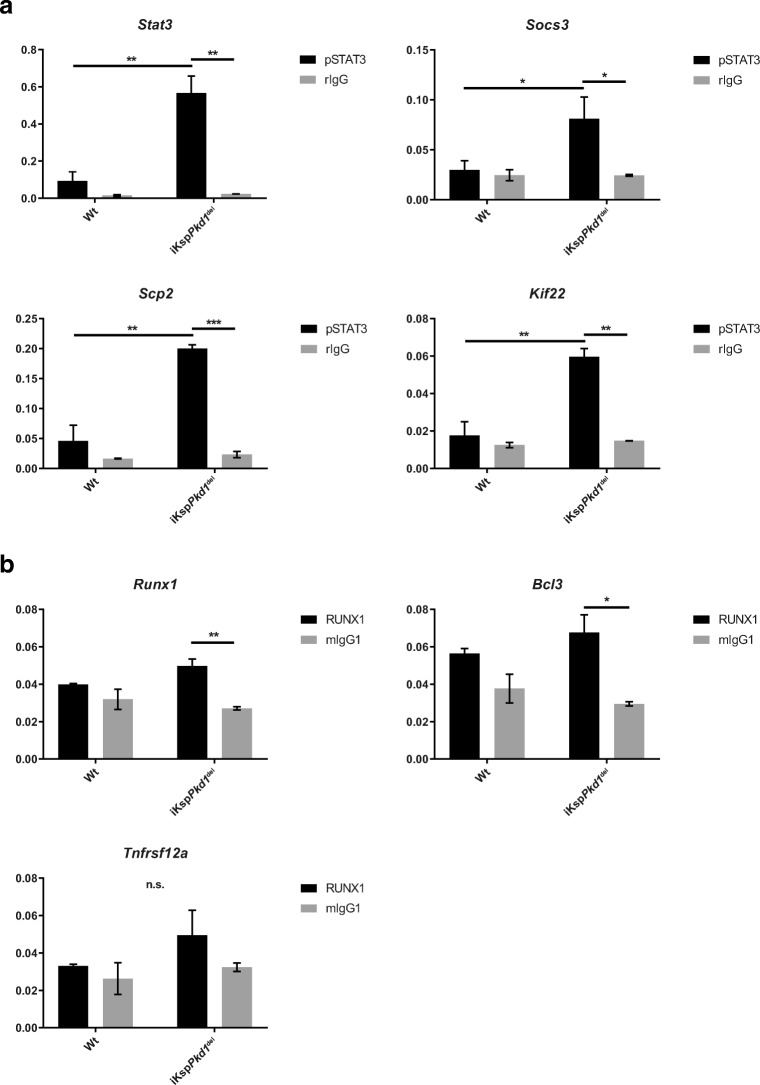
Increased binding of STAT3 and RUNX1 to the promoter of target genes in cystic kidneys, shown by ChIP-qPCR. ChIP-qPCR analysis of end-stage renal disease iKsp*Pkd1*^del^ kidneys or Wt kidneys at 24 weeks after DCVC. **a** We confirmed an increased enrichment for STAT3 binding at target genes in iKsp*Pkd1*^del^ kidneys compared to Wt kidneys. **b** RUNX1 enrichment at its targets is not detected in Wt samples (no difference between RUNX1 ChIP and IgG ChIP) but detected in iKsp*Pkd1*^del^ samples. Black bars pSTAT3 or RUNX1 antibody, grey bars isotype IgG control (rIgG, rabbit IgG; mIgG, mouse IgG). The Y-axis shows the input-normalised binding-enrichment of the TFs to the indicated genomic region. Data represent the mean of two independent ChIPs ± SD; Two-way ANOVA with Tukey’s multiple comparisons test. *P value < 0.05; **P value < 0.01; ***P value < 0.001

As expected, we observed a significantly increased abundance of STAT3 at *Stat3*, *Socs3*, *Scp2* and *Kif22* promoter regions in iKsp*Pkd1*^del^ mice compared to Wt (Fig. 6a, more severe iKsp*Pkd1*^del^ + DCVC and Supplementary Fig. 4a, milder iKsp*Pkd1*^del^ + PBS).

RUNX1 enrichment in iKsp*Pkd1*^del^ mice was not significantly higher than in Wt mice. However, RUNX1 enrichment was significantly higher compared to IgG at the promoter region of *Runx1* and *Bcl3* in iKsp*Pkd1*^del^ mice but not in Wt. A similar trend is observed for *Tnfrsf12a*. This means that in iKsp*Pkd1*^del^ mice, RUNX1 binding is specific, while in Wt, it is not different from the background signal. Thus, RUNX1 is actively binding its targets in cystic kidneys only (Fig. 6b, more severe iKsp*Pkd1*^del^ + DCVC and Supplementary Fig. 4b, milder iKsp*Pkd1*^del^ + PBS).

Overall, these data, in addition to the altered expression levels, show that the activity of STAT3 and RUNX1 is increased in advanced stages of PKD in mice.

### Expression of TFs in kidneys of ADPKD patients

**Fig. 7:**
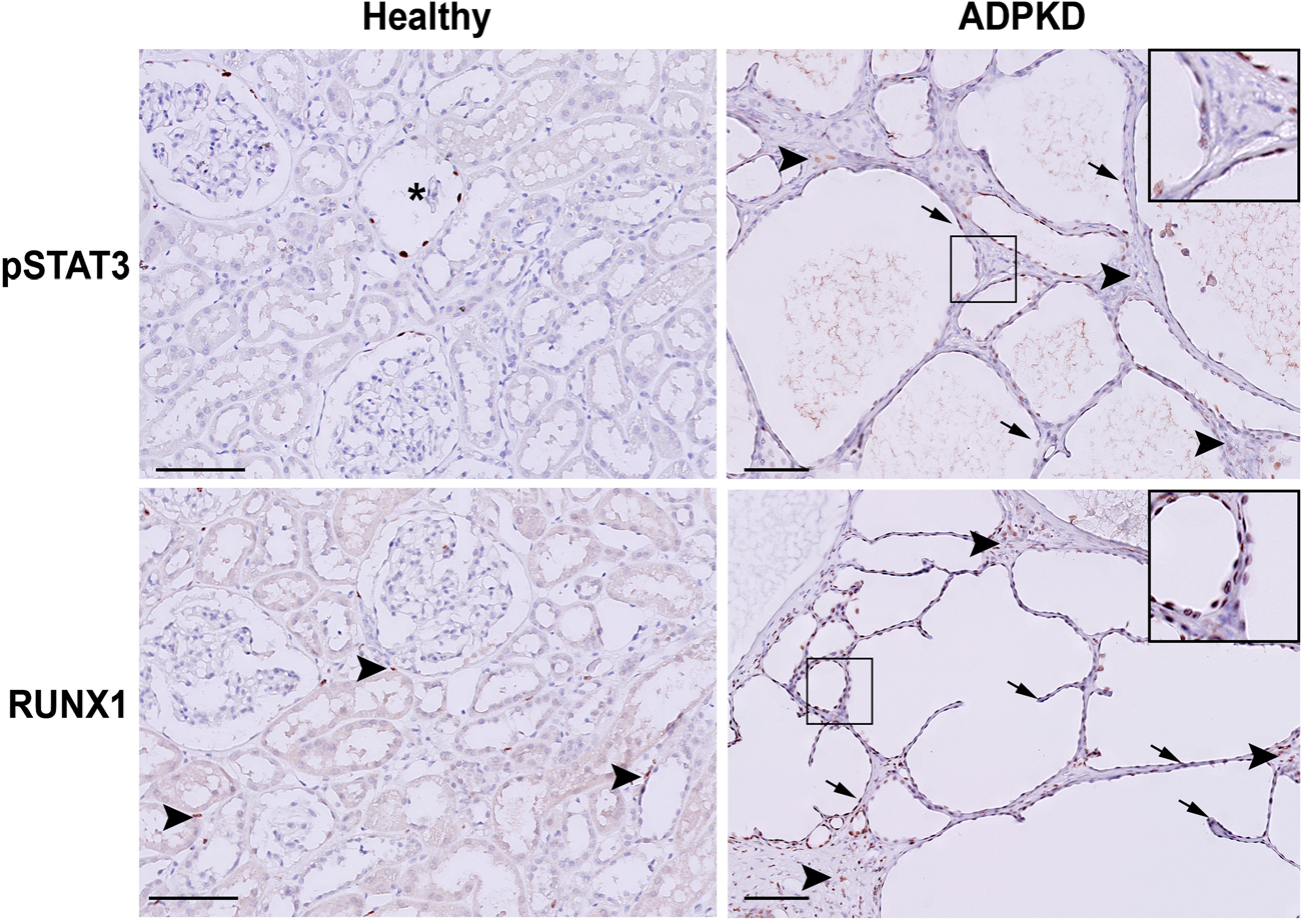
pSTAT3 and RUNX1 expression in human kidneys with ADPKD. Representative immunohistochemistry of human kidneys. In healthy patients, the expression of pSTAT3 and RUNX1 was rarely detected (asterisks). In end-stage cystic kidneys from ADPKD patients, pSTAT3 and RUNX1 localised in the nuclei of the tubular epithelial cells (arrows) and infiltrating cells (arrowheads). Scale bars 100 μm

Lastly, we checked the expression of STAT3 and RUNX1 in human kidney sections obtained from ADPKD patients and healthy controls. Comparably with what was observed in mice, in healthy controls, we found only sporadic expression of pSTAT3 in the nuclei of tubular epithelial cells (Fig. 7, asterisks) and expression of RUNX1 in some infiltrating cells (Fig. 7, arrowheads). Conversely, in ADPKD patients’ renal tissue, the expression of pSTAT3 and RUNX1 was increased in the nuclei of the epithelial cells and infiltrating cells (Fig. 7, right panel and Supplementary Fig. 5, right panel).

These data suggest that the TFs identified by our meta-analysis using rodent models are relevant for human ADPKD.

## Discussion

Previously, we identified a list of 1515 genes dysregulated during PKD progression, which we defined as PKD Signature. We also showed a consistent overlap (about 35%) of the PKD Signature with genes normally involved in injury/repair mechanisms [5]. Now, we have put this analysis a step further by identifying and characterising TFs involved in ADPKD progression.

Using MSigDB, we identified 92 TFs in the PKD Signature and again showed that about 35% of these genes (32 out of 92) have a strong injury-related component. This is in line with a substantial body of literature indicating that injury is a significant modifier in PKD and a potential trigger of cyst formation. Indeed, renal injury causes faster cystic disease progression suggesting that events activated during the injury/repair phase are also crucial for cyst initiation and expansion [3, 4]. Moreover, cyst formation per se is a source of injury for the surrounding tissue making the two pathological processes challenging to dissect [19].

Among these 92 identified TFs, we observed known players in PKD, such as STAT3 [17, 20], c-MYC [18], SMAD2 [21], GLIS2 [22], c-JUN [23] and E2F1 [24], confirming our approach. On the other hand, we did not find TFs such as PPARα, which has been described to play a role in PKD [25]. This is likely due to the high stringency used for the definition of the PKD Signature, which allows us to get specific targets while possibly losing others [5].

Interestingly, we also identified many other TFs, never described before in PKD. Some of these TFs, such as EGR1, KLF5 and FOXM1, have been reported in literature for their involvement in injury/repair mechanisms or pathways dysregulated during PKD progression and might be interesting candidates for future studies. Indeed, *Egr1* is an early growth response gene and is downstream of the mitogen-activated protein kinase (MAPK) pathway, a pathway dysregulated in PKD [23]. EGR1 is a key regulator of proliferation, apoptosis and inflammation and was shown to be involved in renal injury and fibrosis. *Egr1* disruption protected mice from renal failure in a model of tubulointerstitial nephritis and resulted in lower activation of the TGF-β pathway [26]. Moreover, *Egr1* can be downregulated by curcumin, a compound able to reduce cyst formation in vivo [17]. Also, KLF5 was shown to play a role in renal inflammation and fibrosis since unilateral ureteral obstruction in mice haploinsufficient for *Klf5* resulted in reduced renal injury, fibrosis and infiltrating cells [27]. Thus, modulation of KLF5 activity might improve the pro-fibrotic and pro-inflammatory phenotype observed especially during the more advanced phases of PKD progression. *Foxm1* is expressed during cell proliferation and is critical for cell cycle progression. In adult tissues *Foxm1* expression is low, but after injury, its levels are dramatically increased. In particular, FOXM1 can control the expression of genes involved in the G2/M transition phase. Cell-cycle arrest in G2/M phase is associated with pro-fibrotic cytokines production by proximal tubular cells [28]. Not surprisingly, these three TFs are involved in PKD since aberrant extracellular matrix (ECM) deposition is commonly found in PKD patients and animal models of PKD, not only in ESRD but also in early stage [29]. This suggests that increased ECM deposition may be contributing to cyst formation and not barely be a consequence of it, as shown for laminin-alpha5 [30] and integrins-beta1 [31], which mutation could affect the cystic phenotype. Thus, modulation of pro-fibrotic processes could be a valuable strategy to modulate PKD progression.

EGR1, KLF5 and FOXM1, together with ESR1 and STAT3, were also among the significantly enriched PKD Signature TFs identified based on their target genes annotated in the ChEA 2016 database. Pathway analysis of the targets of these TFs, using Genetrail2 and Wikipathways, revealed enrichment for pathways known to play a role in PKD progression, such as the TGF-β pathway, oxidative stress, cellular metabolism, interleukins signalling, adipogenesis, estrogen signalling and apoptosis [21, 32–35]. Using this approach, we also identified TFs not directly present in the PKD Signature. Interestingly, the top five TFs identified based on their targets were all described in literature to be involved in the progression of PKD (STAT3)[17, 20, 36] or in processes relevant for PKD like angiogenesis (E2F7)[37], DNA damage response (E2F7, TRIM28)[38, 39], renal injury and fibrosis (EGR1)[26], epithelial cell proliferation, apoptosis and adhesion (TP63)[40]. Nevertheless, apart from STAT3, the TFs themselves had never been associated with PKD before and therefore could be interesting subjects for future studies. Surprisingly, we did not find back RUNX1 in this list as the level of enrichment was just below the significance threshold (data not shown). Nevertheless, we confirmed increased expression and activity of RUNX1 during PKD progression in mice and human ADPKD kidneys. Thus, we speculate that the absence of RUNX1, as well as other TFs potentially involved in PKD, is due to limitations related with the ChEA database, such as the source of ChIP-data, the way the different studies have been analysed and the actual TFs included in the database.

To further test and validate our approach, we selected for additional wet-lab validation STAT3 and RUNX1 as they showed the most significant change in expression both in PKD progression and injury. By performing ChIP-qPCR for STAT3 and RUNX1 in ADPKD-affected kidneys, we confirmed increased transcriptional activity in cystic kidneys for these TFs. Persistent activation of STAT3 has been described in several mouse models for ADPKD as well as in human cystic tissues [17, 20, 36]. STAT3 usually is not active in adult kidneys but is abundantly present, suggesting that it can be readily activated at needs, such as after injury [36]. Indeed, STAT3 activation has been shown in several different mouse models with renal injury [41, 42]. Thus, the fact that we found back STAT3 and several of its putative targets in our signature proved the reliability of our meta-analysis.

RUNX1 involvement in ADPKD has never been described before. RUNX1 is one of the Runt domain TFs, together with RUNX2 and RUNX3. RUNX2 expression has been shown to be regulated by PC1 in osteoblasts, proving the existence of an interaction between the two proteins [43]. Nevertheless, expression of RUNX2 or RUNX3 is not increased after injury nor during disease progression in murine (cystic) kidneys (RNA-Seq data identifier E-MTAB-5319 published in Malas et al*.,* 2017 [5]). RUNX1 is expressed in the epithelium of several organs during development, among which the kidneys [44]. It participates in the regulation of cell cycle, cell proliferation and apoptosis [45] and has been described in several models for lung, muscle and brain injury [46–48]. Recently, a study was published suggesting that RUNX1 is an important regulator of TGF-β-induced renal tubular epithelial-to-mesenchymal transition (EMT) and fibrosis [49]. As mentioned above, TGF-β signalling is involved in ECM deposition and cyst progression and is partly responsible for the EMT observed in cystic kidneys. Modulation of TGF-β-related signalling is associated with amelioration of the cystic phenotype [21]. Thus, it is plausible that RUNX1 might play a role in ADPKD progression. In fact, inhibition of STAT3 signalling with more or less specific inhibitors, such as curcumin, pyrimethamine and S3I-201, has been proven to improve the cystic phenotype in different mouse models [17, 20, 36]. Similarly, we propose that targeting RUNX1, for example, using microRNAs as described for prostate cancer [50], or other molecular or pharmacological approaches, might also result in amelioration of the cystic phenotype.

We observed increased expression of STAT3 and RUNX1 also after injury in Wt mice, suggesting that these TFs orchestrate injury/repair mechanisms and that increased expression is not necessarily related to *Pkd1* deletion. Notably, dissecting PKD progression and injury is not easy, since injury can speed up cyst initiation/growth, which in turn causes injury to the surrounding tissue. Therefore, it is plausible that both STAT3 and RUNX1 are facilitating PKD progression by activating injury/repair pathways normally inactive in fully developed and healthy kidneys.

To conclude, our comprehensive analyses identified a signature of TFs differentially expressed in PKD and to a certain extent also in injury/repair. Several of these TFs are involved in processes able to support cyst formation and progression, nevertheless were never described before in PKD, suggesting that they might be interesting targets for therapy. However, further analyses are needed to identify the molecular pathways that these TFs modulate to contribute to PKD progression and cyst formation. Additionally, the TFs we identified are a subset of the TFs involved in PKD and not a comprehensive list. This is due to limitations in the annotation databases we used and RNA-Seq technology. To establish a comprehensive list of TFs involved in PKD and/or injury, further studies must be conducted on protein levels and protein phosphorylation status. That said, our approach was capable of robustly identifying 92 TFs, and additional wet-lab validations confirmed the involvement of RUNX1 and STAT3 making this paper a starting point to understand the role of TFs in PKD progression.

## Electronic supplementary material

Table S1 (XLSX 40 kb)

Table S2 (XLSX 63 kb)

Table S3 (XLSX 18 kb)

Supplementary Fig. 1 ADPKD mouse model with kidney injury and PKD progression. Experimental pipeline and data partly presented in Formica et al. [15] **a** Experimental pipeline. Adult mice (around 14 weeks old) were treated with tamoxifen to induce *Pkd1* deletion. One week after gene inactivation, mice were injected with the nephrotoxic compound DCVC and sacrificed at 1, 2, 5 and 10 weeks after DCVC and when the mice reached end-stage renal disease, indicated by blood urea nitrogen level (BUN) over 25 mmol/l. **b** BUN of Wt and iKsp*Pkd1*^del^ mice showing increased BUN at 40 h after DCVC injection (t-test, *P* value < 0.0001). BUN levels are back to baseline at 1 week after DCVC and remain at a physiological level up to 5 weeks after DCVC injection (t-test, not significant). Each point is the mean of 6 mice ± SD. **c** Representative histology of Wt and iKsp*Pkd1*^del^ mice before and after injury. At 1 week, it is possible to observe mild tubule dilation in both Wt and iKsp*Pkd1*^del^ mice which are largely resolved at 2 weeks. Scale bar 50 μm. **d** In Wt mice BUN is in a physiological range up to 24 weeks after DCVC injection when the mice were sacrificed. The iKsp*Pkd1*^del^ mice injected with DCVC (red solid line) reach end-stage renal disease earlier compared to PBS-treated mice (light-blue dashed line). Median DCVC group, 14 weeks; median PBS group, 19 weeks; n = 6, Mann-Whitney test, *P* value < 0.05. **e** Representative histology of Wt and iKsp*Pkd1*^del^ kidneys. At 10 weeks after DCVC, iKsp*Pkd1*^del^ mice show tubule dilation and small cyst spread over the kidneys, which are absent in the PBS-treated group or in the Wt mice. At kidney failure, iKsp*Pkd1*^del^ kidneys show evident cyst formation while the Wt kidneys show no aberration in both groups with or without injury. Scale bar 1 mm

Supplementary Fig. 2 Overview of pSTAT3 and RUNX1 expression in Wt and iKsp*Pkd1*^del^ mice after injury and during cyst progression. **a** Low magnification of Wt and iKsp*Pkd1*^del^ kidneys at 1 week after DCVC (+ injury) or PBS (– injury). With this magnification, it is possible to appreciate that the expression of pSTAT3 and RUNX1 in non-injured kidneys was present mainly in some interstitial cells, while after injury, the expression was clearly visible in the nuclei of the epithelial cells (brown nuclei). In particular, tubules in the corticomedullary region, which are more sensitive to the toxic insult, showed the most staining. **b** Low magnification of Wt and iKsp*Pkd1*^del^ kidneys at 10 weeks after DCVC (“10weeks”; left and middle panel) and at kidney failure (“KF”; right panel) when the kidneys are severely cystic. With this magnification, it is visible that Wt and normal-looking tissue in mutant mice (mildly cystic kidneys at “10weeks”) showed expression of pSTAT3 and RUNX1 mainly in some interstitial cells, while cyst-lining epithelial cells, epithelial cells of surrounding tubules and infiltrating cells showed clear nuclear pSTAT3 and RUNX1 staining. Scale bars 100 μm

Supplementary Fig. 3 Gene and protein expression of the TFs in cells. **a** Gene expression of *Stat3* and *Runx1* in mIMCD3 cells (n = 3). On the Y-axis, we show the TFs expression normalised on the geometric mean of two housekeeping genes, *Ywhaz* and *Rplp0*. **b** In the middle panel, western blot is showing the protein expression of RUNX1 (about 50 kDa) and GAPDH (about 37 kDa) in Jurkat cells (used as a positive control) and two renal epithelial cell lines, mIMCD3 and PTEC. In the right panel, quantification of the western blot normalised on GAPDH expression is shown. Low but visible RUNX1 expression is observed in both renal epithelial cell lines.

Supplementary Fig. 4 Enrichment of STAT3 and RUNX1 at their targets in Wt and iKsp*Pkd1*^del^ mice treated with PBS. ChIP-qPCR analysis of end-stage renal disease iKsp*Pkd1*^del^ kidneys (median 21 weeks after PBS, equals age 8 months) or Wt kidneys (24 weeks after PBS, equals age 9 months). **a** We confirmed an increased enrichment for STAT3 at the promoter region of their target genes. **b** RUNX1 enrichment at its targets is not detected in Wt samples but show a trend in iKsp*Pkd1*^del^ samples. The Y-axis shows the input-normalised binding-enrichment of the TFs to the indicated genomic region. Data represent the mean of two independent ChIPs ± SD; Two-way ANOVA with Tukey’s multiple comparisons test. *P value < 0.05; **P value < 0.01; ***P value < 0.001 (JPG 709 kb)

Supplementary Fig. 5 Overview of pSTAT3 and RUNX1 expression in human kidneys with ADPKD. **a** Low magnification of healthy and ADPKD affected human kidneys showing that in healthy kidneys the expression of pSTAT3 and RUNX1 was present mainly in some interstitial cells, while in ADPKD kidneys cyst-lining epithelial cells, epithelial cells of surrounding tubules and infiltrating cells showed clear nuclear pSTAT3 and RUNX1 staining (brown nuclei). Scale bars 100 μm (JPG 1891 kb)
