# Supplementary figures and images for: Characterisation of transcription factor profiles in polycystic kidney disease (PKD): identification and validation of STAT3 and RUNX1 in the injury/repair response and PKD progression
Source: J Mol Med (Berl). 2019 Nov 26;97(12):1643–56. doi: 10.1007/s00109-019-01852-3 (PMC6920240; doi:10.1007/s00109-019-01852-3)

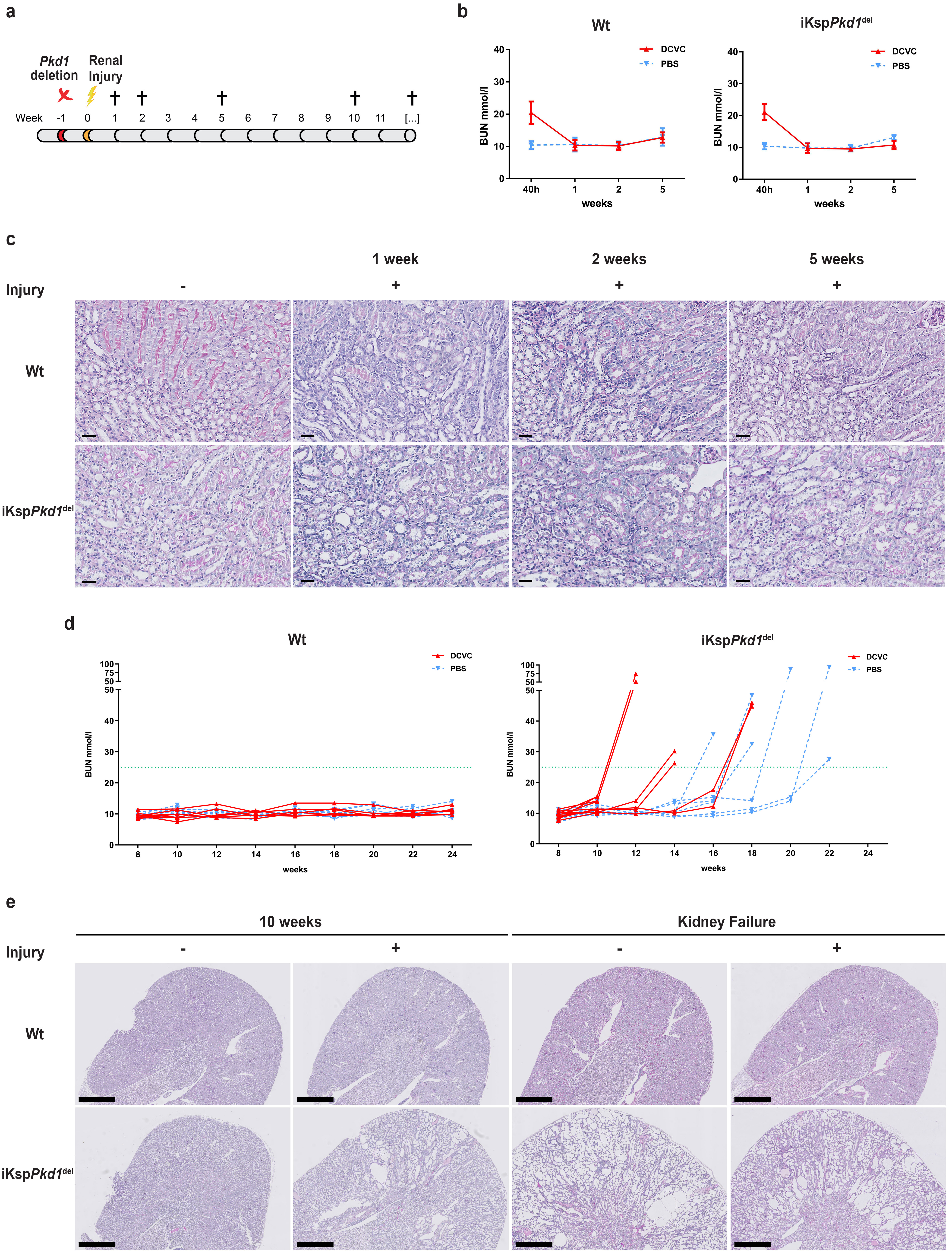

Supplement: Supplementary file 4 — ADPKD mouse model with kidney injury and PKD progression. Experimental pipeline and data partly presented in Formica et al. [15] a Experimental pipeline. Adult mice (around 14 weeks old) were treated with tamoxifen to induce Pkd1 deletion. One week after gene inactivation, mice were injected with the nephrotoxic compound DCVC and sacrificed at 1, 2, 5 and 10 weeks after DCVC and when the mice reached end-stage renal disease, indicated by blood urea nitrogen level (BUN) over 25 mmol/l. b BUN of Wt and iKspPkd1del mice showing increased BUN at 40 h after DCVC injection (t-test, P value < 0.0001). BUN levels are back to baseline at 1 week after DCVC and remain at a physiological level up to 5 weeks after DCVC injection (t-test, not significant). Each point is the mean of 6 mice ± SD. c Representative histology of Wt and iKspPkd1del mice before and after injury. At 1 week, it is possible to observe mild tubule dilation in both Wt and iKspPkd1del mice which are largely resolved at 2 weeks. Scale bar 50 μm. d In Wt mice BUN is in a physiological range up to 24 weeks after DCVC injection when the mice were sacrificed. The iKspPkd1del mice injected with DCVC (red solid line) reach end-stage renal disease earlier compared to PBS-treated mice (light-blue dashed line). Median DCVC group, 14 weeks; median PBS group, 19 weeks; n = 6, Mann-Whitney test, P value < 0.05. e Representative histology of Wt and iKspPkd1del kidneys. At 10 weeks after DCVC, iKspPkd1del mice show tubule dilation and small cyst spread over the kidneys, which are absent in the PBS-treated group or in the Wt mice. At kidney failure, iKspPkd1del kidneys show evident cyst formation while the Wt kidneys show no aberration in both groups with or without injury. Scale bar 1 mm [file 109_2019_1852_MOESM4_ESM.jpg]

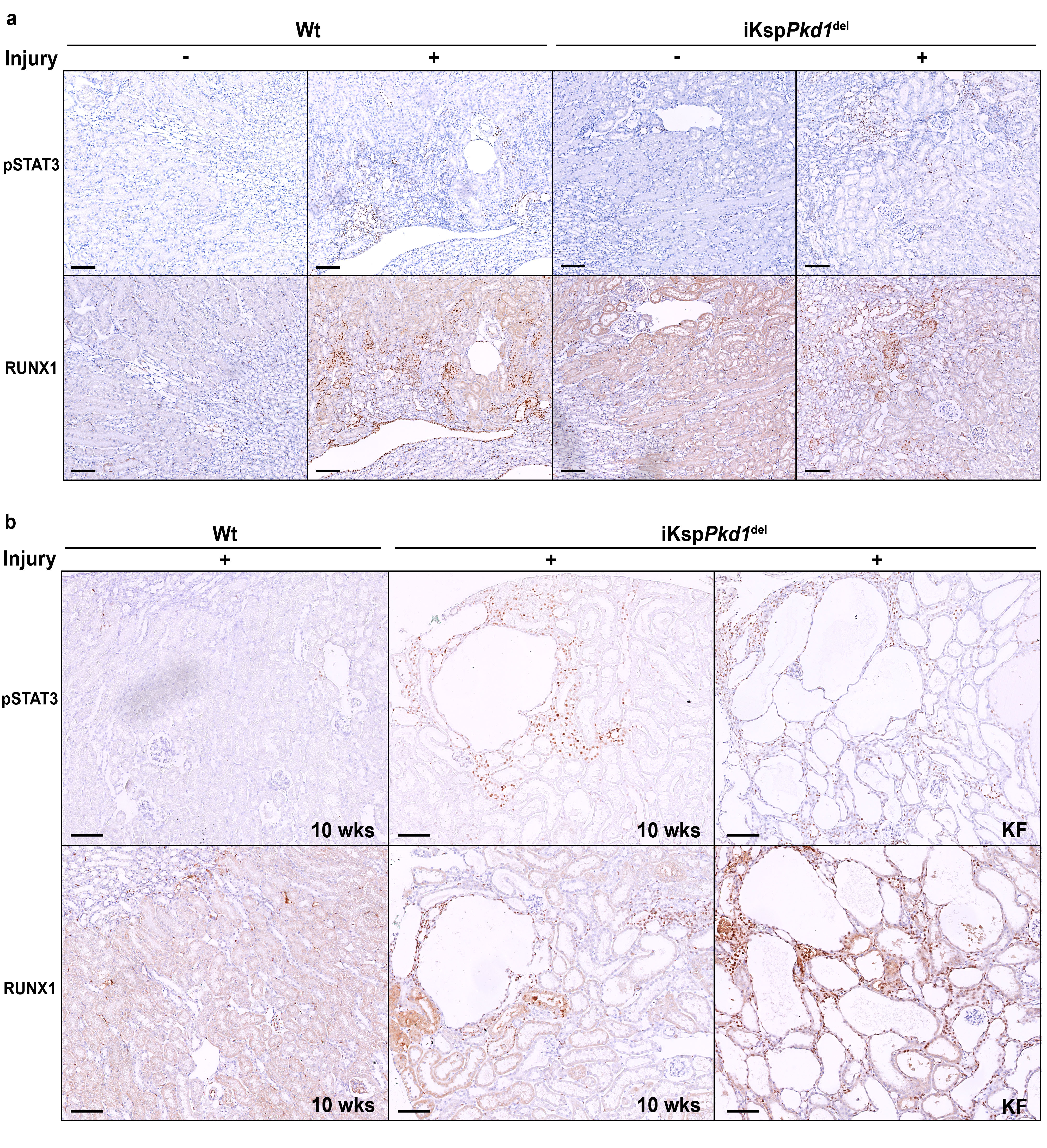

Supplement: Supplementary file 5 — Overview of pSTAT3 and RUNX1 expression in Wt and iKspPkd1del mice after injury and during cyst progression. a Low magnification of Wt and iKspPkd1del kidneys at 1 week after DCVC (+ injury) or PBS (– injury). With this magnification, it is possible to appreciate that the expression of pSTAT3 and RUNX1 in non-injured kidneys was present mainly in some interstitial cells, while after injury, the expression was clearly visible in the nuclei of the epithelial cells (brown nuclei). In particular, tubules in the corticomedullary region, which are more sensitive to the toxic insult, showed the most staining. b Low magnification of Wt and iKspPkd1del kidneys at 10 weeks after DCVC (“10weeks”; left and middle panel) and at kidney failure (“KF”; right panel) when the kidneys are severely cystic. With this magnification, it is visible that Wt and normal-looking tissue in mutant mice (mildly cystic kidneys at “10weeks”) showed expression of pSTAT3 and RUNX1 mainly in some interstitial cells, while cyst-lining epithelial cells, epithelial cells of surrounding tubules and infiltrating cells showed clear nuclear pSTAT3 and RUNX1 staining. Scale bars 100 μm [file 109_2019_1852_MOESM5_ESM.jpg]

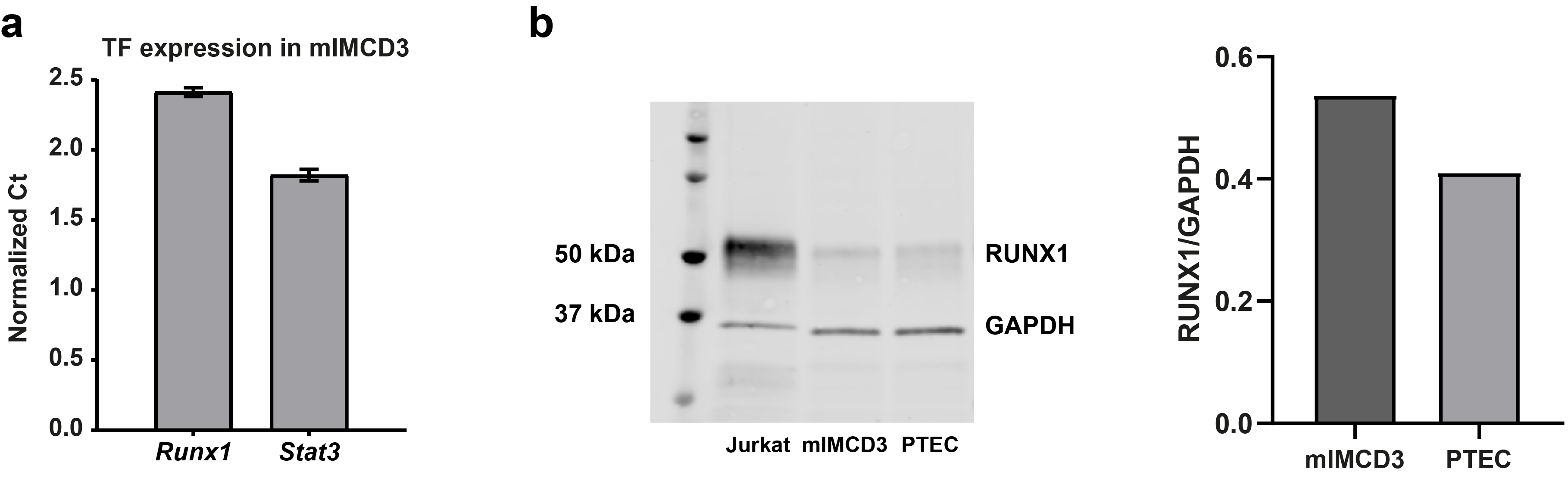

Supplement: Supplementary file 6 — Gene and protein expression of the TFs in cells. a Gene expression of Stat3 and Runx1 in mIMCD3 cells (n = 3). On the Y-axis, we show the TFs expression normalised on the geometric mean of two housekeeping genes, Ywhaz and Rplp0. b In the middle panel, western blot is showing the protein expression of RUNX1 (about 50 kDa) and GAPDH (about 37 kDa) in Jurkat cells (used as a positive control) and two renal epithelial cell lines, mIMCD3 and PTEC. In the right panel, quantification of the western blot normalised on GAPDH expression is shown. Low but visible RUNX1 expression is observed in both renal epithelial cell lines. [file 109_2019_1852_MOESM6_ESM.jpg]

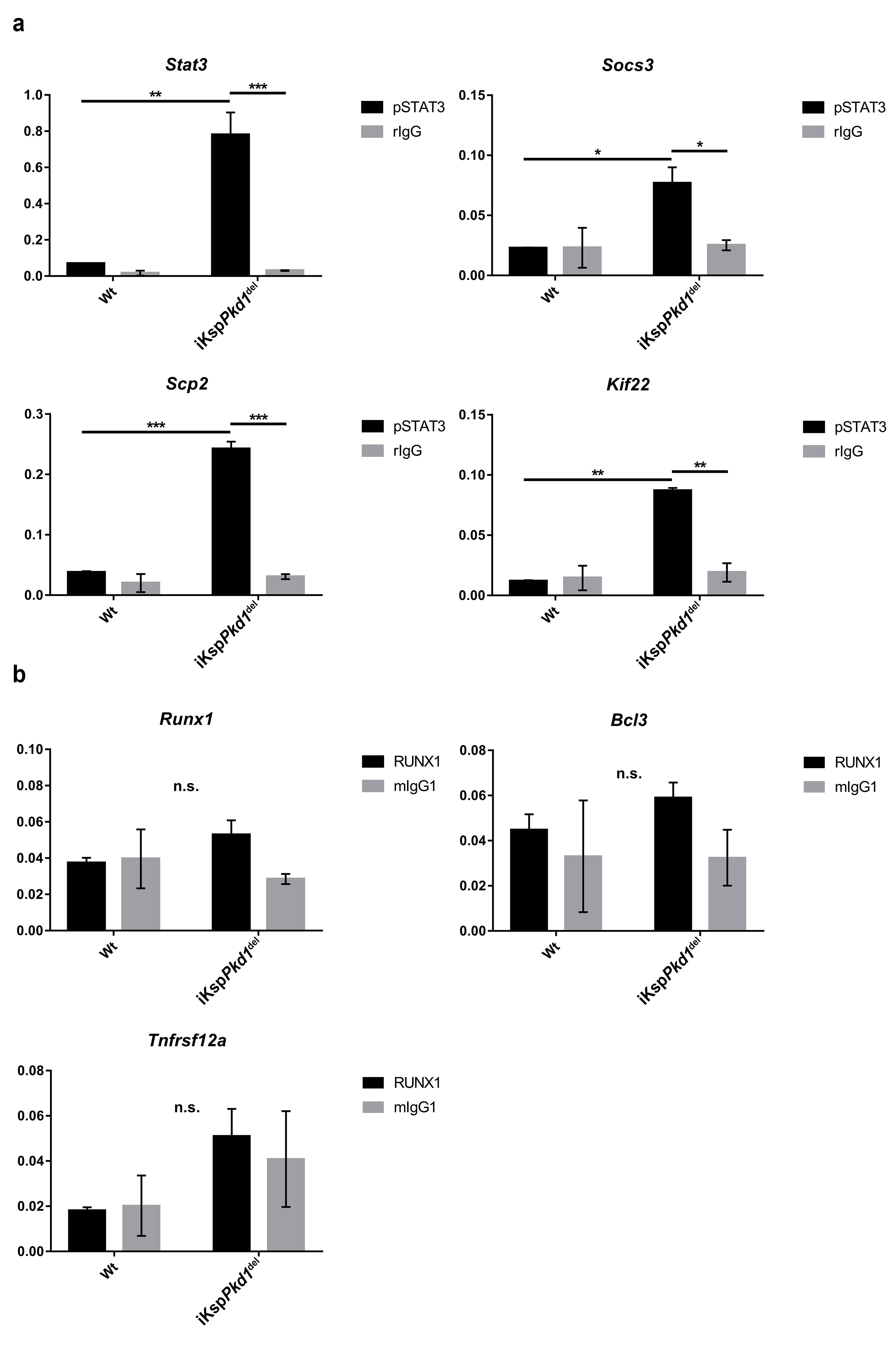

Supplement: Supplementary file 7 — Enrichment of STAT3 and RUNX1 at their targets in Wt and iKspPkd1del mice treated with PBS. ChIP-qPCR analysis of end-stage renal disease iKspPkd1del kidneys (median 21 weeks after PBS, equals age 8 months) or Wt kidneys (24 weeks after PBS, equals age 9 months). a We confirmed an increased enrichment for STAT3 at the promoter region of their target genes. b RUNX1 enrichment at its targets is not detected in Wt samples but show a trend in iKspPkd1del samples. The Y-axis shows the input-normalised binding-enrichment of the TFs to the indicated genomic region. Data represent the mean of two independent ChIPs ± SD; Two-way ANOVA with Tukey’s multiple comparisons test. *P value < 0.05; **P value < 0.01; ***P value < 0.001 (JPG 709 kb) [file 109_2019_1852_MOESM7_ESM.jpg]

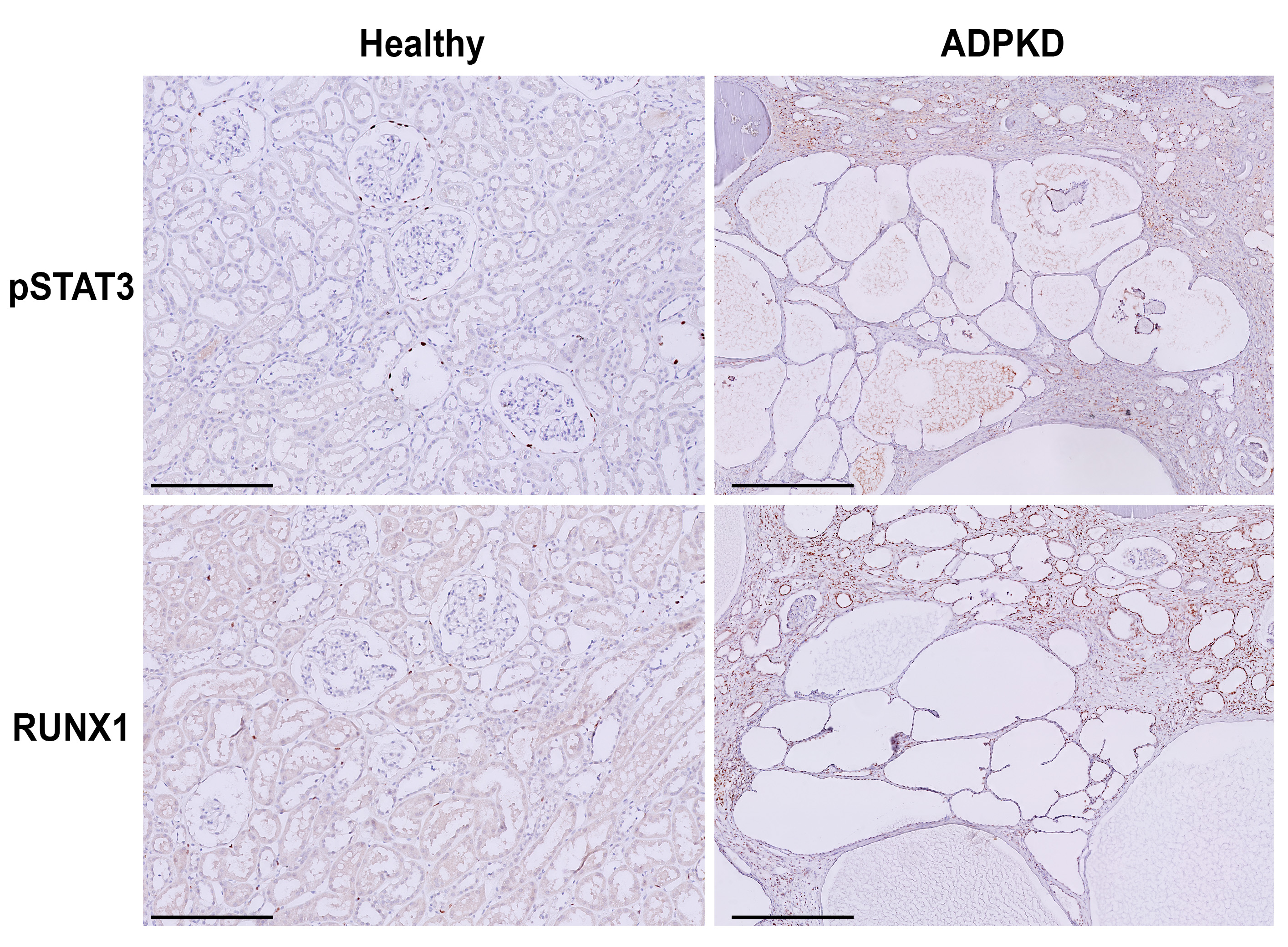

Supplement: Supplementary file 8 — Overview of pSTAT3 and RUNX1 expression in human kidneys with ADPKD. a Low magnification of healthy and ADPKD affected human kidneys showing that in healthy kidneys the expression of pSTAT3 and RUNX1 was present mainly in some interstitial cells, while in ADPKD kidneys cyst-lining epithelial cells, epithelial cells of surrounding tubules and infiltrating cells showed clear nuclear pSTAT3 and RUNX1 staining (brown nuclei). Scale bars 100 μm (JPG 1891 kb) [file 109_2019_1852_MOESM8_ESM.jpg]
